# Molecular Phylogeny, Character Evolution, and Biogeography of *Hydrangea* Section *Cornidia*, Hydrangeaceae

**DOI:** 10.3389/fpls.2021.661522

**Published:** 2021-06-29

**Authors:** Carolina Granados Mendoza, Esteban Manuel Martínez Salas, Paul Goetghebeur, Stefan Wanke, Marie-Stéphanie Samain

**Affiliations:** ^1^Departamento de Botánica, Instituto de Biología, Universidad Nacional Autónoma de México, Mexico City, Mexico; ^2^Research Group Spermatophytes, Ghent University, Gent, Belgium; ^3^Institut für Botanik, Technische Universität Dresden, Dresden, Germany; ^4^Herbario Nacional de México, Departamento de Botánica, Instituto de Biología, Universidad Nacional Autónoma de México, Mexico City, Mexico; ^5^Ghent University Museum, Botanical Garden, Ghent University, Gent, Belgium; ^6^Red de Diversidad Biológica del Occidente Mexicano, Centro Regional del Bajío, Instituto de Ecología, A.C., Pátzcuaro, Michoacán, Mexico; ^7^Systematic and Evolutionary Botany Lab, Department of Biology, Ghent University, Gent, Belgium

**Keywords:** ancestral area, Asia, functional dioecism, disjunct distribution, hortensias, long-distance dispersal, neotropics

## Abstract

**Background:** *Hydrangea* section *Cornidia* consists of 26 currently accepted species and a yet undefined number of new species and erroneously synonymized taxa. This clade consists of (sub)tropical lianas occurring from northern Mexico to southern Chile and Argentina, and one species from Southeast Asia. Currently, no molecular phylogenetic hypothesis is available that includes more than a few species of this section. Hence, a resolved and well-sampled molecular phylogenetic hypothesis may help to enforce taxonomic decisions. In this study, we present a phylogenetic framework based on sequences from two low copy nuclear genes from a comprehensive taxon sampling of *H*. section *Cornidia* and a selection of outgroups. Our phylogenetic reconstructions prove the non-monophyly of the traditionally recognized subsections *Monosegia* and *Polysegia* and their corresponding series, *Speciosae* and *Aphananthae*, and *Synstyleae* and *Chorystyleae*, respectively. Three morphologically defined species were recovered with high support as monophyletic, namely, *Hydrangea panamensis, Hydrangea serratifolia*, and *Hydrangea tarapotensis*. However, statistical support for some shallow nodes did not allow to refute, with high support, the monophyly of several of the herein recognized species for which more than one individual could be analyzed. Based on the obtained phylogenetic framework, we reconstructed the evolution of selected reproductive characters. *Hydrangea* section *Cornidia* is the only genus section for which dioecism has been extensively documented. Our character reconstruction of sexual dimorphism shows that dioecism is the ancestral state in this section and that this was reversed to monoecy in *Hydrangea seemannii* and *Hydrangea integrifolia*. Character reconstruction for the enlarged marginal flowers recovered their presence as the ancestral character state in *H*. section *Cornidia*, although at least three internal lineages independently lost them; thus, losses were reconstructed to be more likely than gain. With respect to the flower color, more species exhibit white than red flowers, and white is reconstructed as the ancestral state. *Cornidia* also shows an unusual disjunct geographic distribution between Asia and Central Mesoamerica—South America, as it is not present in the USA and Canada. The origin of *Cornidia* is reconstructed to be the New World with higher probability, and the presence of one species in Asia is likely due to long-distance dispersal.

## Introduction

Hortensias are well-known ornamental shrubs, rarely lianas, with an Asian or North American origin (Assocation des Amis de la Collection Nationale d' Hydrangea Shamrock, 2001; Samain et al., 2010). A recent infrageneric classification (De Smet et al., 2015), which is based on genetic data, grouped eight satellite genera in a broadly circumscribed genus *Hydrangea*, maintaining the familiar generic names as sections, as molecular data rendered traditional *Hydrangea* polyphyletic. One of the least known sections is *Hydrangea* L. section *Cornidia* Ruiz and Pav., consisting of lianas that occur from northern Mexico to southern Chile and Argentina with one species, *Hydrangea integrifolia* Hayata, in southeastern Asia. Most of the species grow at higher elevations (1,000–3,000 m) at tropical and subtropical latitudes, favoring temperate climates, generally occurring in the cloud forest or in the ecotone between cloud forest and tropical rainforest. This section consists of 26 currently accepted species, as well as a yet undefined number of potentially new species and taxa that have been erroneously synonymized (Samain et al., 2014, 2019, 2021; Samain and Martínez Salas, 2015). All taxa of *Hydrangea* section *Cornidia* (hereafter shortened as *Cornidia*) are evergreen root climbers growing up to 60 m high in the canopy of mostly primary forests, or rarely on boulders and rock walls (Granados Mendoza et al., 2014), generally functionally dioecious, rarely monoecious, with opposite coriaceous leaves and hortensia-like whitish, greenish, yellowish, reddish, or purplish inflorescences, with or without enlarged marginal flowers (Samain et al., 2019, 2021).

*Cornidia* has been shown to be monophyletic, including the single Asian species (i.e., *H. integrifolia*; Jacobs, 2010; Samain et al., 2010; Granados Mendoza, 2013; Granados Mendoza et al., 2013, 2015; De Smet et al., 2015) and is sister to *Hydrangea* section *Calyptranthe* Maxim., consisting of climbing species with Asian origin (De Smet et al., 2015). Both clades together are sister to *Hydrangea* section *Asperae* (Rehder) Y. De Smet and Samain, encompassing Asian shrubby species (De Smet et al., 2015).

**Figure 1:**
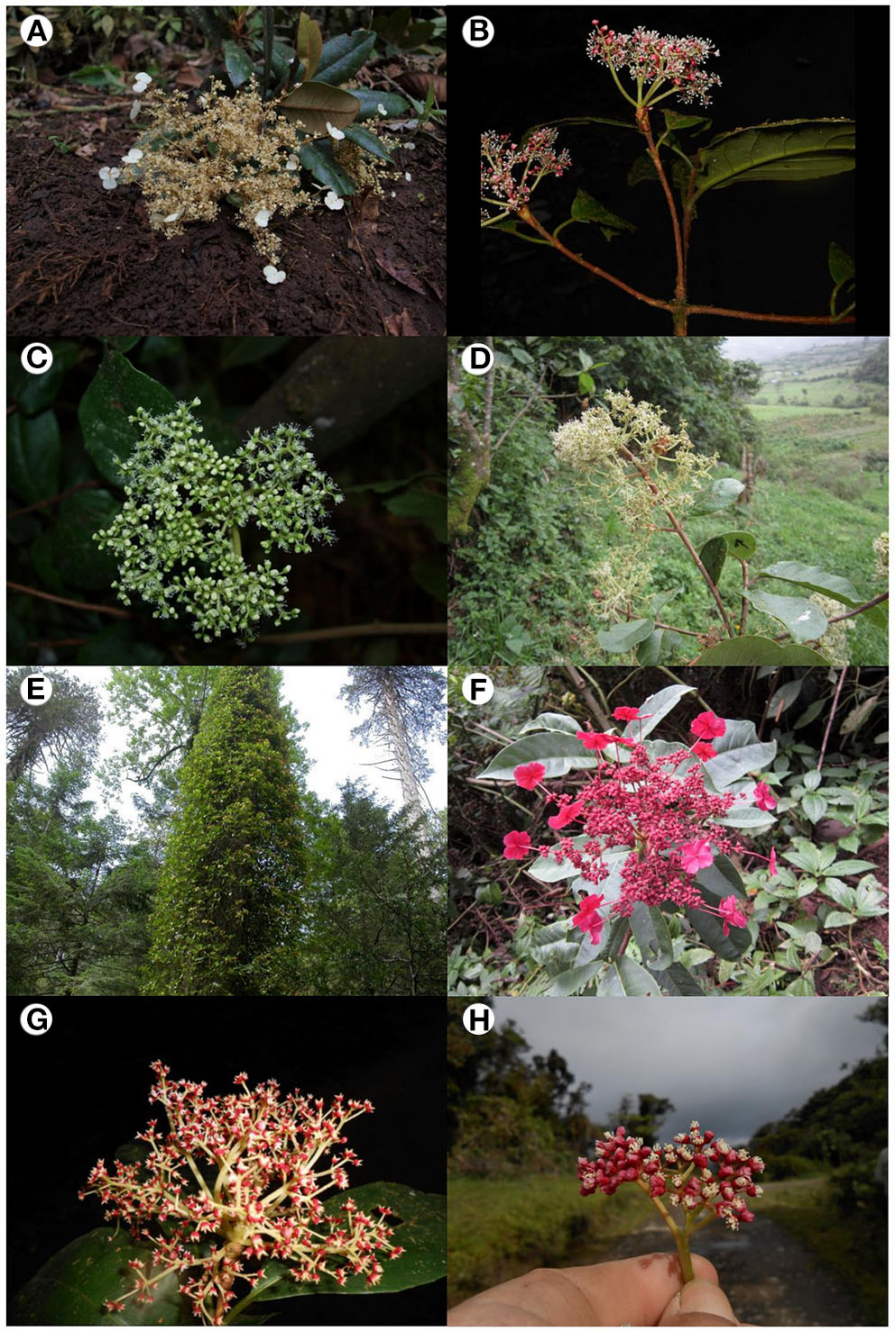
Representative species of *Hydrangea* section *Cornidia*: **(A)** *Hydrangea asterolasia*, simple inflorescence with male flowers (stamens large, pistils reduced) and enlarged marginal flowers (Ecuador, Granados Mendoza et al., 2012-042, clade J); **(B)** *H. diplostemona*, simple inflorescence with male flowers (stamens large, pistils reduced) without enlarged marginal flowers (Costa Rica, Samain, and Martínez Salas 2013-029, clade G); **(C)** *H. ecuadorensis*, simple inflorescence with female flowers (pistils large, anthers reduced) without enlarged marginal flowers (Ecuador, Granados Mendoza et al., 2012-067, clade E); **(D)** *H. jelskii*, branched inflorescence with male flowers (stamens large, pistils reduced) without enlarged marginal flowers (Peru, Samain et al., 2011-063, clade F); **(E)** *H. seemannii*, flowering plant growing on coniferous tree (Mexico, Granados Mendoza et al., 483, clade A); **(F)** *H. weberbaueri*, simple infructescence with immature fruits (Peru, Samain et al., 2011-062, epitype, clade H); **(G)** *H. diplostemona*, simple inflorescence with female flowers (pistils large, stamens reduced) without enlarged marginal flowers (Costa Rica, Samain and Martínez Salas, 2013-028, clade G); and **(H)** *H. oerstedii*, central cyme of inflorescence (enlarged marginal flowers not included) with female flowers (pistils large, stamens reduced) (Costa Rica, Samain and Martínez Salas, 2013-022, clade D). Clade names correspond to those discussed in the text.

The extensive morphological variation in *Cornidia* was implicitly acknowledged for in the morphological descriptions in previous studies (e.g., McClintock, 1957; Freire-Fierro, 2004; Christenhusz, 2009), which is reflected by highly variable characters within particular species, and this, in turn, led to a further going synonymization of names, as species in this clade were considered highly variable. As a consequence, the more than 40 species that had been described since the description of the genus *Cornidia* by Ruiz and Pavón (1794) were synonymized into 11 accepted species by 2009, not taking into account the many diagnostic morphological characters. We have carried out extensive fieldwork throughout the distribution area of *Cornidia* since 2009. Therefore, we realized, on the one hand, that its representatives are much more common than previously thought and, on the other hand, that its immense morphological variation did not fit into the then 11 accepted species. Although just a handful of species can be characterized by a synapomorphic morphological character, all taxa defined in the ongoing work are easily recognizable by a unique combination of characters. Additionally, intraspecific variation is usually relatively limited, with the exception of species with a more widespread distribution or certain differences between female and male plants of a single species (Figure 1).

Given that the majority of the species of *Cornidia* are threatened by extinction due to habitat destruction and climate change (Samain et al., 2014, 2019, 2021), the delimitation of units for conservation and identification of their specific threats are urgent. We have recently described seven new species for Mexico (Samain et al., 2014, 2019), and the ongoing study of Hydrangeas of Central and South America includes both the description of species new to science and the preparation of amended descriptions of taxa, which do fit within earlier described species that had been erroneously synonymized (Samain et al., 2021).

Given the abovementioned variation in this group, we expect *Cornidia* to consist of approximately 50–60 species, some widespread, others with rather limited distribution. Within this updated taxonomic framework, we can finally intend to unravel their intricate biogeographic and evolutionary patterns. Simultaneously, a resolved and well-supported phylogenetic hypothesis may help us to answer taxonomic questions as well as to enforce taxonomic decisions.

The aims of the present *Cornidia* study are (1) to provide a first molecular phylogenetic hypothesis based on a highly representative sampling, (2) to test former taxonomic subdivision of the section and the monophyly of morphologically defined entities, (3) to reconstruct the evolution of key floral morphological characters, and (4) to identify the biogeographic origin of the section. These main objectives will help to understand the evolution of Hydrangeaceae, particularly in the Neotropics.

## Methods

### Taxon Sampling

Fieldwork has been carried out by the authors in Chile, Costa Rica, Ecuador, Mexico, and Peru, during which leaf tissue samples of individuals were collected in silica gel for DNA isolation. We have collected extensively in the three most diverse Neotropical countries with respect to *Hydrangea*, which in order of decreasing species number are: Ecuador, Peru, and Mexico. These are followed by Colombia, where we were not yet able to collect due to administrative reasons, and Costa Rica, where we have explored and sampled repeatedly. In the case of Colombia, there are about 10 endemic species that have not been collected recently, and 2–3 even are known from the type collection only (Samain et al., 2021, unpublished data).

The focal group was represented by 89 individuals corresponding to 21 currently recognized species (Samain et al., 2014, 2019, 2021), 11 species that will be recognized (pending publication of ongoing taxonomical studies), and six or seven undescribed taxa, representing about 60–70% of the expected total species diversity in this clade (Supplementary Material 1). Our sampling within *Cornidia* includes the most comprehensive taxonomical, morphological, and geographical representation of this group to date. Herbarium material of species that we did not have access to during fieldwork generally could not be included due to insufficient DNA quality, as it concerned specimens that were not recently collected. Nevertheless, the present sampling can be considered to be highly representative from a geographical point of view and allows to elucidate the ancestral geographic areas of the different clades at the macrolevel. Besides 88 *Cornidia* accessions representing the North, Central, and South American distribution, one accession of *H. integrifolia* of Southeast Asia was included. *Hydrangea anomala* of section *Calyptranthe*, the sister lineage of *Cornidia*, and six species of section *Asperae*, the sister lineage to the clade composed of sections *Cornidia* and *Calyptranthe* (De Smet et al., 2015), were included as outgroup. The species *Hydrangea quercifolia* W. Bartram was used to root the tree since it was recovered by Granados Mendoza et al. (2013) in a grade with section *Hydrangea* (i.e., *H. arborescens* L.) and the clade composed of sections *Asperae, Calyptranthe*, and *Cornidia*. A total of 97 individuals were sequenced, all corresponding to species belonging to the clade “Hydrangea I” sensu Samain et al. (2010) and De Smet et al. (2015; Supplementary Material 1).

### Molecular Markers

Portions of two low-copy nuclear genes were chosen based on their reported utility for solving shallow divergence in *Hydrangea* (*TIF3H1* gene; Granados Mendoza et al., 2015) or in angiosperms, in general (*SMC1* gene; Zhang et al., 2012), as well as for their ease of amplification and sequencing on the herein included taxon sampling. The amplified portion of the *TIF3H1* gene (Translation initiation factor 3 subunit H1, further referred to as *TIF3H1* gene) is located between the exons VI and XI of its *Arabidopsis thaliana* ortholog *AT1G10840*, whereas the one corresponding to the *SMC1* gene (structural maintenance of chromosomes 1, further referred to as *SMC1* gene) is flanked by the exons XIV and XVI of its *A. thaliana* ortholog *AT3G54670*.

### Molecular Methods

Extraction of genomic DNA was performed from silica dried leaf material, using the DNeasy Plant Mini Kit (Qiagen GmbH) or a standard CTAB method (Doyle and Doyle, 1987). Amplification of the *TIF3H1* gene was performed in two partially overlapping pieces (ca. 217 bp of overlap), using the primer combination H-AT1G10840-2F: 5′-TTCAAGGTCCAACCAAGGTGTCTTA-3′/H-AT1G10840-2R: 5′-TTGCTGCTGGGCTTGCTGAC-3′ for the first one, and H-AT1G10840-3F: 5′-CAACTATCAACCAATCCATT-3′/H-AT1G10840-3R: 5′-TGATTTGTTATGAGAAAACTATCC-3′ for the second. In some cases, a third primer combination (H-AT1G10840-2F/H-AT1G10840-3R) was used to close gaps between the amplified portions by the former two primer combinations. The *SMC1* gene was also amplified in two pieces that overlap ca. 1,000 bp with the primer combinations H-SMC1-16F: 5′-GAAGAKTGTGARAAGGAMATA-3′/H-SMC1-18R: 5′-TGTGTTGCTYTTKGTAAGTTGTTT-3′ for the first piece, and H-SMC1-17F: 5′-TGGACCAATACGAGGCTTTG-3′/H-SMC1-18R for the second. PCR reaction mix included 8 μl dNTP (Carl Roth + Co.KG, 1.25 mM each), 2 μl of each primer (10 pmol/μl), 10 μl Taq buffer (GoTaq® Reaction Buffer, 7.5 mM MgCl2), 0.25 μl of Taq DNA polymerase (GoTaq® DNA Polymerase, 5 u/μl), 0.5–2 μl of DNA template, and water added accordingly to obtain a final volume of 50 μl. Both markers were amplified with a touchup gradient PCR profile (Rowther et al., 2012), which included an initial denaturation at 94°C for 2 min, followed by a loop of 10 cycles repeated five times consisting of denaturation at 96°C for 45 s, annealing at a temperature gradient (starting 5°C below the optimal annealing temperature for each primer pair and increasing 0.5°C every cycle) for 30 s and extension at 72°C for 1 min/kb of the expected length. A final extension at 72°C for 7 min was applied. PCR products were run on a 1.2% agarose gel and subsequently purified using a gel extraction kit (Macherey and Nagel). Sequencing was performed through the Macrogen Europe sequencing service.

### Sequence Edition, Alignment, and Phylogenetic Hypotheses Reconstruction

Sequence contigs were produced and edited in Geneious R9 v.9.1.8 (https://www.geneious.com). The online version of MAFFT v.7 with the default settings was used for initial alignments, which were subsequently inspected and adjusted by hand. One 6-bp long ambiguously aligned region corresponding to poly-T and poly-A in the *SMC1* gene was excluded for further analyses. Both the original alignment and the alignment excluding the abovementioned ambiguously aligned region can be found in the Supplementary Material 2. Maximum likelihood (ML) phylogenetic analyses were performed on a combined matrix containing the *TIF3H1* and *SMC1* genes, as well as on two individual matrices of the *TIF3H1* and *SMC1* genes, respectively. Each of these three matrices was partitioned by exonic and intronic regions, and PartitionFinder2 v.2.1.1 (Lanfear et al., 2017) was used to determine the best-fit subset partitioning scheme. One hundred independent searches for the best tree were run for each matrix, and node support was estimated through 1,000 bootstrap replicates on RAxML v.8.2.10 (Stamatakis, 2014). TreeGraph2 v.2.14.−771 (Stöver and Müller, 2010) was used for collapsing nodes with BS <50, and trees were further edited with FigTree v.1.4.4 (Rambaut, 2018).

### Floral Character Evolution

The evolution of three discrete floral characters was reconstructed: (1) functional flower sex (functionally dioecious or monoecious), (2) presence of marginal flowers (yes or no), and (3) flower color (purple, red, or white; this color refers to the sepals of the enlarged marginal flowers and the petals of the reduced flowers). Characters were coded based on a detailed study of the field collections performed since 2009, as well as the herbarium material. Species with unknown character states were pruned from the respective tree with the pruning tool of RASP v.4.2 (Yu et al., 2015). A Bayesian stochastic character mapping approach (Nielsen, 2001; Huelsenbeck et al., 2003) was used for character-state history reconstruction on the combined ML tree. The *make.simmap* function of the R package *phytools* (Revell, 2012) was used with 1,000 simulations and an “all transition rates different” (ARD) model (Revell, 2012). No prior distributions of nodes were applied. For ease of visualization, node probabilities were drawn on the ML tree converted to a cladogram.

### Ancestral Area Reconstruction

The R (https://www.R-project.org/) package BioGeoBEARS v.1.1.1 (Matzke, 2013) was used for reconstruction of ancestral areas of geographic distribution in RASP v.4.2 (Yu et al., 2015). The best ML tree obtained was uploaded together with a distribution area-coded matrix. Distribution areas were coded as follows: northwestern Mexico (a), Central and Mesoamerica (b), northern South America (c), southern South America (d), Asia (e), and southeastern United States of America (f). Species distribution was coded based on extensive field observations performed since 2009, as well as herbarium studies. It is worth mentioning that, given the very large distribution area of *Cornidia*, we delimited and coded large areas for analysis, as, at this stage of our study of this group, we are focusing on understanding macropatterns rather than more detailed models on a microscale. Area delimitation is based on observations of distribution and endemism patterns of the section. Selection of model of the geographic range was performed in a likelihood framework, setting the number of maximum unit areas allowed in ancestral distributions to two. The Akaike's information criterion (AIC) and the likelihood-ratio test with a significant *p*-value of 0.01 were used to select the best fit model. Models evaluated included the Dispersal–Extinction–Cladogenesis (DEC), Dispersal–Vicariance Analysis (DIVA), and Bayesian inference of historical biogeography for discrete areas (BayArea), each of them with and without their variant for founder event speciation with long-distance dispersal +J. The +J parameter was evaluated in model comparison because all *Cornidia* species possess dust-like wind-dispersed seeds, suggesting that a founder-event speciation factor might play a role in the biogeographic history of this group.

## Results

### Phylogenetic Relationships

**Figure 2:**
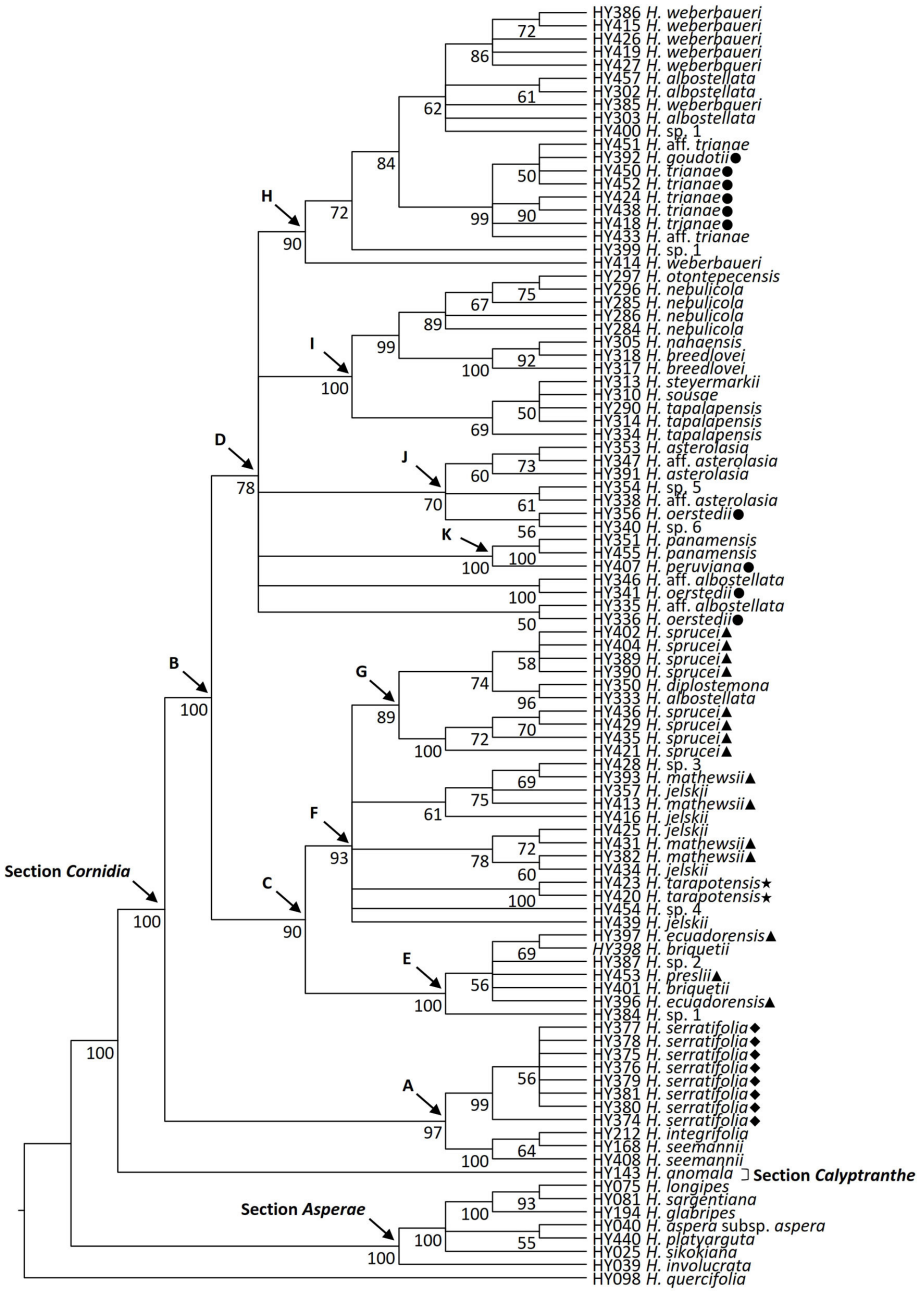
Phylogenetic relationships of *Hydrangea* section *Cornidia* based on the combined analysis of the *SMC1* and *TIF3H1* low-copy nuclear genes DNA sequences. Bootstrap values ≥50% are shown below the branches. Current sectional classification (*sensu* De Smet et al., 2015) is denoted in boldface. Shapes in front of species names indicate Briquet's (1919) infrasectional classification as follows: subsection *Monosegia* series *Speciosae* (circle), subsection *Monosegia* series *Aphananthae* (triangle), subsection *Polysegia* series *Synstyleae* (star), and subsection *Polysegia* series *Chorystyleae* (diamond). A–K denote main clades discussed in the text.

Individual gene trees and the tree from the combined analysis were congruent in almost all the recovered highly supported relationships, except for the position of one *Cornidia* accession (HY414, *Hydrangea weberbaueri*), which was found with high support associated with two different clades in the *TIF3H1* gene and the combined trees (Figure 2, Supplementary Material 3). Phylogenetic relationships will be described based on the tree resulting from the combined analysis where clades with BS <50 have been collapsed (Figure 2). In this tree, the monophyletic section *Asperae* (BS = 100) is in a grade with sections *Calyptranthe* and the monophyletic *Cornidia* (both BS = 100).

Within *Cornidia*, two main lineages were recovered. In the first main lineage (clade A; BS = 97), a clade including all *Hydrangea serratifolia* accessions (BS = 99) is sister to a clade (BS = 100) where two *Hydrangea seemannii* accessions are successive sisters of *Hydrangea integrifolia* (BS = 64). The second main lineage (clade B; BS = 100) is further divided into two lineages (clades C and D; BS = 90 and 78, respectively). Clade C consists of a sister pair in which one clade (clade E; BS = 100) includes one accession of *H*. sp. 1 sister to a polytomy (BS = 56) of one accession each of *Hydrangea ecuadorensis* and *Hydrangea briquetii, Hydrangea preslii, H*. sp. 2, and a clade of two other accessions of *H. briquetii* and *H. ecuadorensis* (BS = 69). The other clade (clade F, BS = 93) consists of a polytomy of one accession of *Hydrangea jelskii*; *Hydrangea* sp. 4; the monophyletic species *Hydrangea tarapotensis* (BS = 100); a clade (BS = 78) including two sister pairs with one accession each of *H. jelskii* and *Hydrangea mathewsii* (BS = 60 and 72, respectively); a clade (BS = 61) including one accession of *H. jelskii*, sister to a polytomy (BS = 75) of one accession each of *H. mathewsii* and *H. jelskii*, and a clade (BS = 69) of one accession of *H. mathewsii* and *H*. sp. 3; and clade G (BS = 89), which is composed of two clades, one including four accessions of *Hydrangea sprucei* (BS = 100) and the other (BS = 74) consisting of a sister pair of one clade of *Hydrangea diplostemona* and one *Hydrangea albostellata* accession (BS = 96) and the other clade of four additional *H. sprucei* accessions (BS = 58). Clade D includes two clades (BS = 50 and 100, respectively), containing one accession of *H*. aff. *albostellata* and of *Hydrangea oerstedii*, each in polytomy with four other clades herein referred to as clades K, J, I and H, described as follows. Within clade K (BS = 100), *Hydrangea peruviana* is sister to the monophyletic *Hydrangea panamensis* (BS = 100). Clade J (BS = 70) is composed of a polytomy of a clade where *H*. sp. 6 is sister to one accession of *H. oerstedii* (BS = 56), a clade where one accession of *H*. aff. *asterolasia* is sister to *H*. sp. 5 (BS = 61), and a clade (BS = 60) where two *H. asterolasia* accessions are successive sisters of one accession of *H*. aff. *asterolasia* (BS = 73). Clade I (BS = 100) is divided into two clades, the first of them (BS = 69) includes one accession of *H. tapalapensis* sister to a clade (BS = 50) where two other accessions of this species are in polytomy with *Hydrangea sousae* and *Hydrangea steyermarkii* and the second clade (BS = 99) includes two lineages: one (BS = 100) where two accessions of *Hydrangea breedlovei* are successive sisters of *Hydrangea nahaensis* (BS = 92) and the other (BS = 89) where two accessions of *Hydrangea nebulicola* are in polytomy, with a clade (BS = 67) of two other accessions of this species: one of them sister to *Hydrangea otontepecensis* (BS = 75). In clade H (BS = 90), *Hydrangea weberbaueri* is in a grade with one accession of *H*. sp. 1 (BS = 72) and a clade (BS = 84) consisting of a sister pair where one clade (BS = 99) is composed of a polytomy of one accession of *Hydrangea* aff. *trianae*, one accession of *H. trianae*, a clade (BS = 90) of two accessions of the latter species, and a clade (BS = 50) where two accessions of *H. trianae* are in polytomy with *Hydrangea goudotii*, and another accession of *H*. aff. *trianae*. The second clade (BS = 62) includes a polytomy of one accession of *H*. sp. 1, *H. albostellata*, one accession of *H. weberbaueri*, a clade of two accessions of *H. albostellata* (BS = 61), and a clade including five accessions of *H. weberbaueri* (BS = 86).

### Evolution of Floral Traits

**Figure 3:**
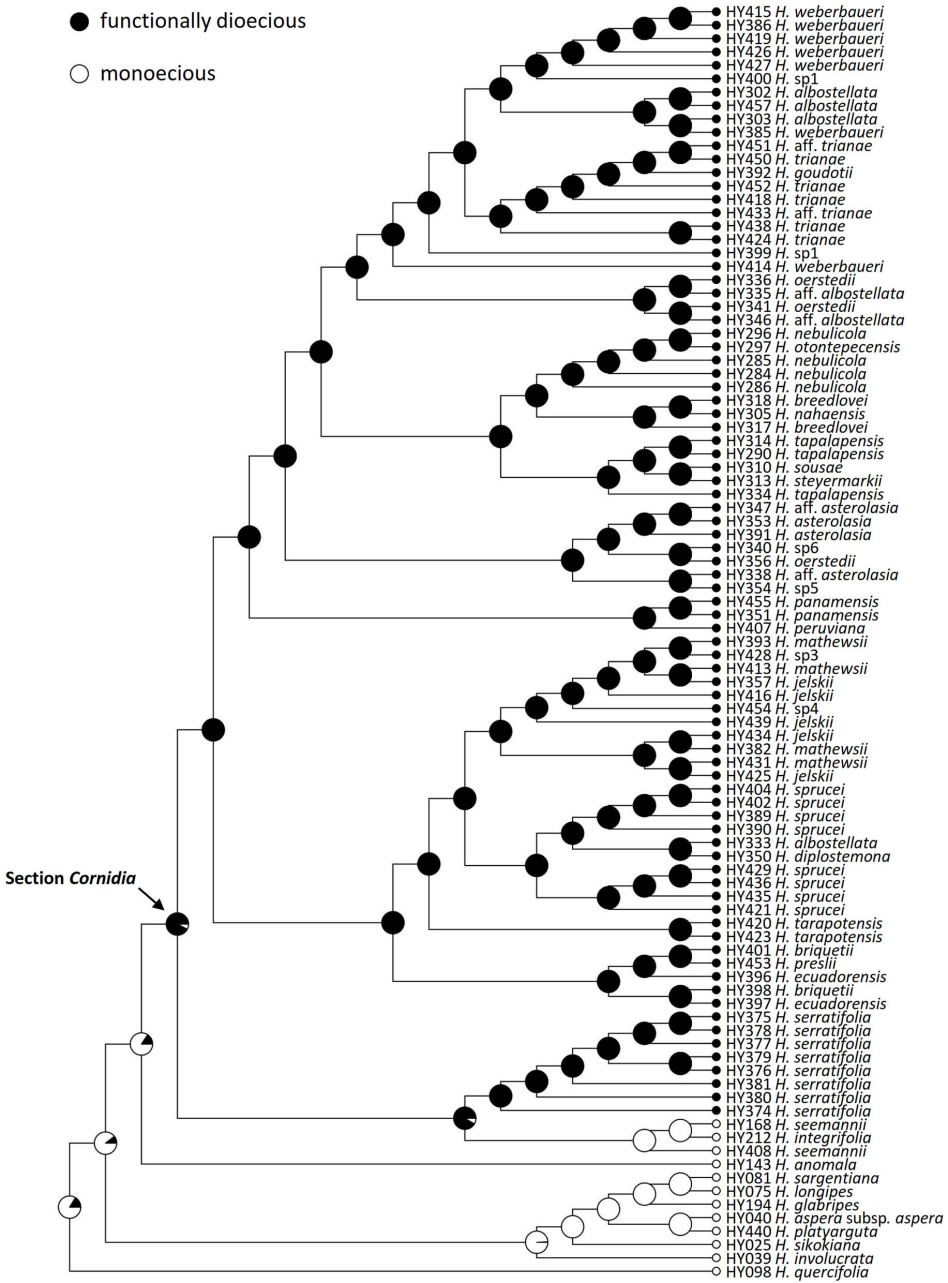
Ancestral reconstruction of sexual dimorphism in *Hydrangea* section *Cornidia*, using Bayesian stochastic character mapping. Pies indicate the proportion of functionally dioecious or monoecious from 1,000 simulations on the phylogeny resulting from the combined analysis.

**Figure 4:**
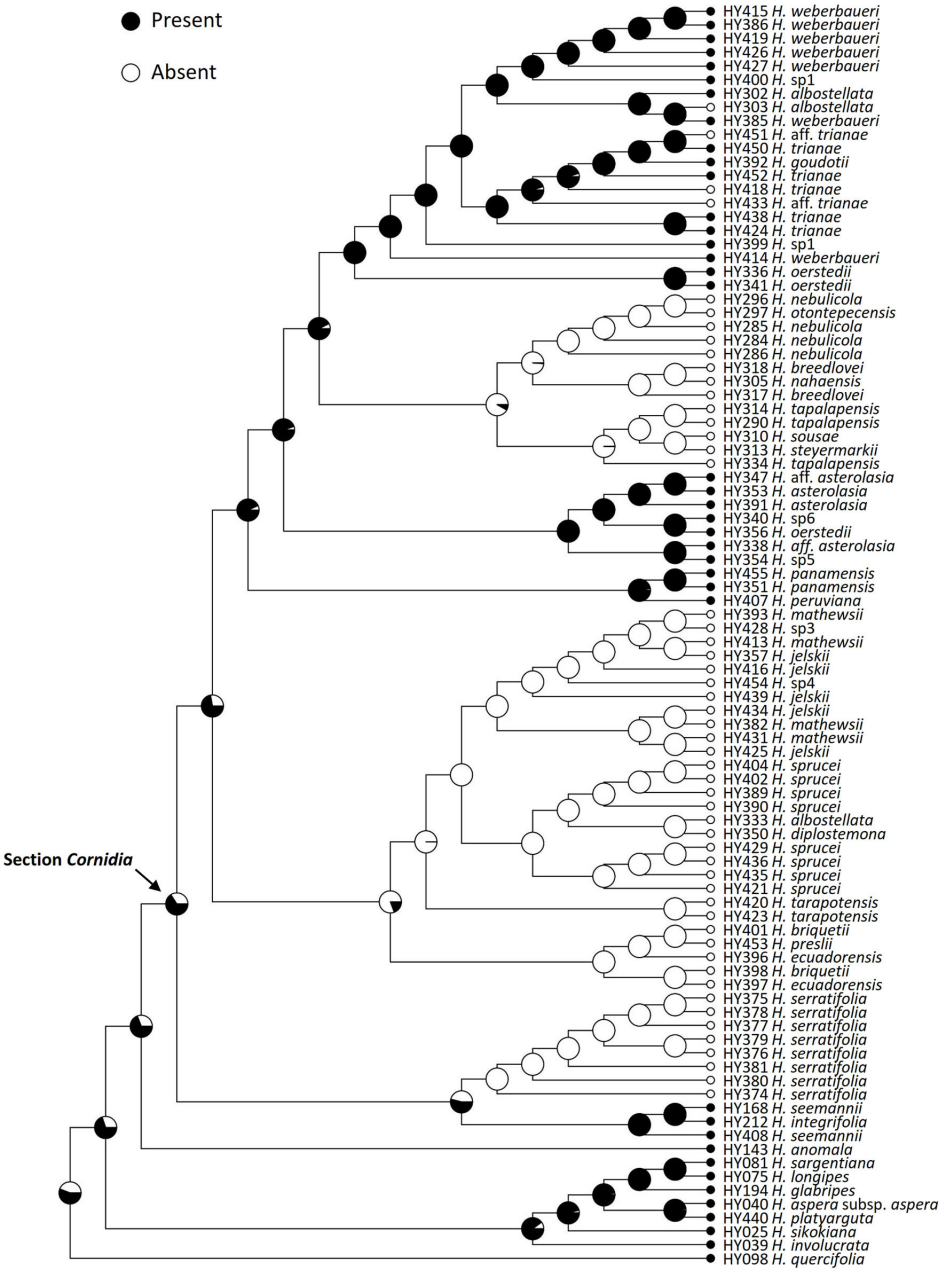
Ancestral reconstruction of the presence of marginal flowers in *Hydrangea* section *Cornidia* using Bayesian stochastic character mapping. Pies indicate the proportion for the presence and the absence of marginal flowers from 1,000 simulations on the phylogeny resulting from the combined analysis.

The stochastic character mapping for the functional flower sex retrieved 2.7 changes on average of both types, where changes from functionally dioecious to monoecious are slightly more frequent (1.563, 95% HPD = 0−4) than in the opposite direction (1.137, 95% HPD = 0−2; Figure 3). Simulations for the presence of marginal flowers recovered on average 15.59 changes of both types, with the loss (9.876, 95% HPD = 6–13) of this structure being more likely than its gain (5.719, 95% HPD = 1–9; Figure 4).

**Figure 5:**
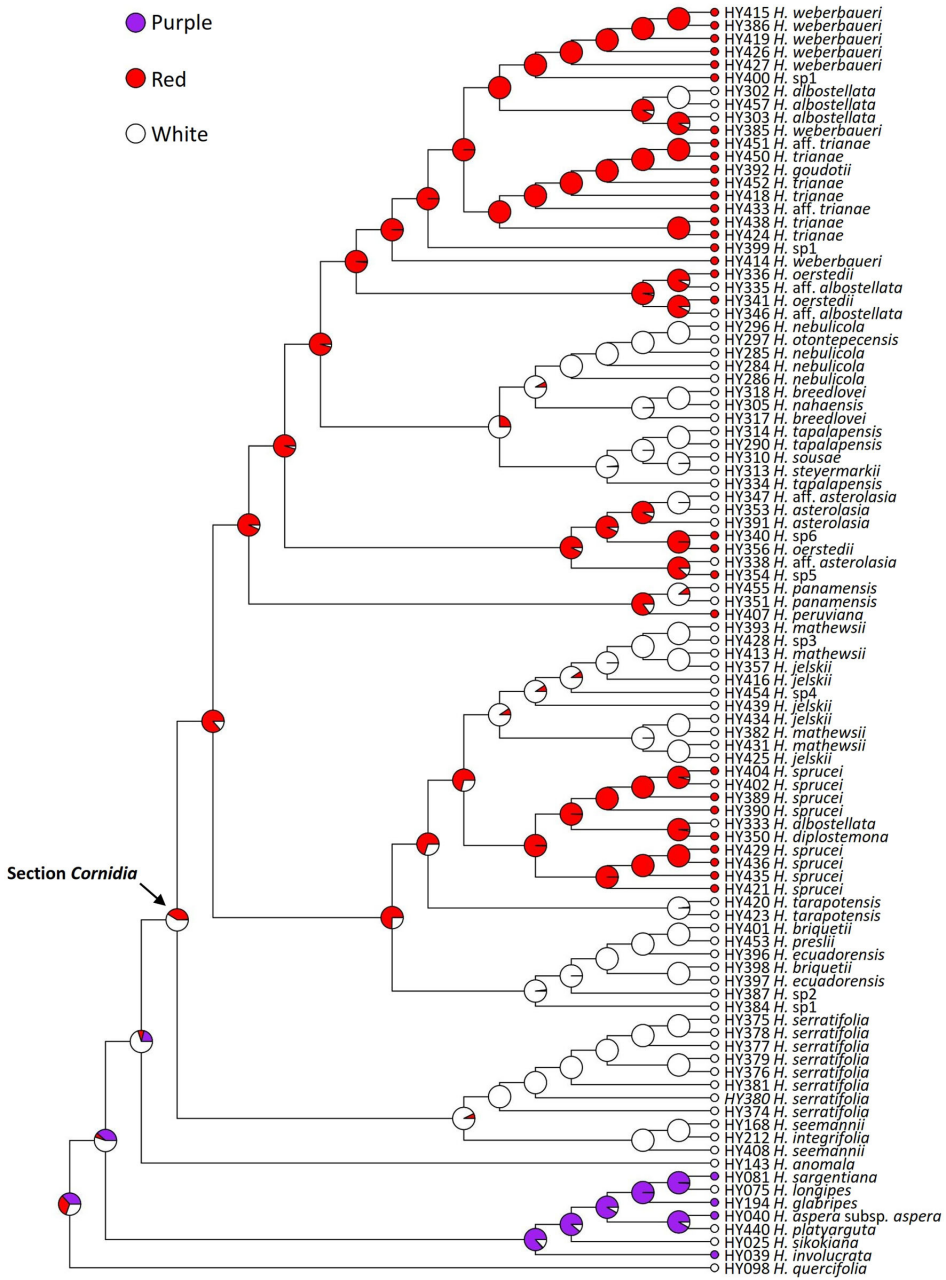
Ancestral reconstruction of flower color in *Hydrangea* section *Cornidia* using Bayesian stochastic character mapping. Pies indicate the proportion for each color from 1,000 simulations on the phylogeny resulting from the combined analysis.

In *Cornidia*, there are more species with white than with red flowers, and white is reconstructed as the ancestral state. Clade A is exclusively composed of species with white flowers. However, both ancestral nodes of clade C and D are reconstructed to have red flowers, although the change from white to red is much less likely than the opposite (Figure 5). Within clades C and D, at least three and four shifts to white occurred, respectively. Flower color simulations showed 29.4 changes among states on average of all types, except those between purple and red. The estimated transition matrix for flower color indicated that the most likely change is red to white (18.011, 95% HPD = 12–23), followed by the transitions purple to white (4.68; 95% HPD = 1–7), white to red (4.939, 95% HPD = 1–8), and white to purple (1.773, 95% HPD = 0−4; Figure 5).

### Biogeographic Reconstruction

**Figure 6:**
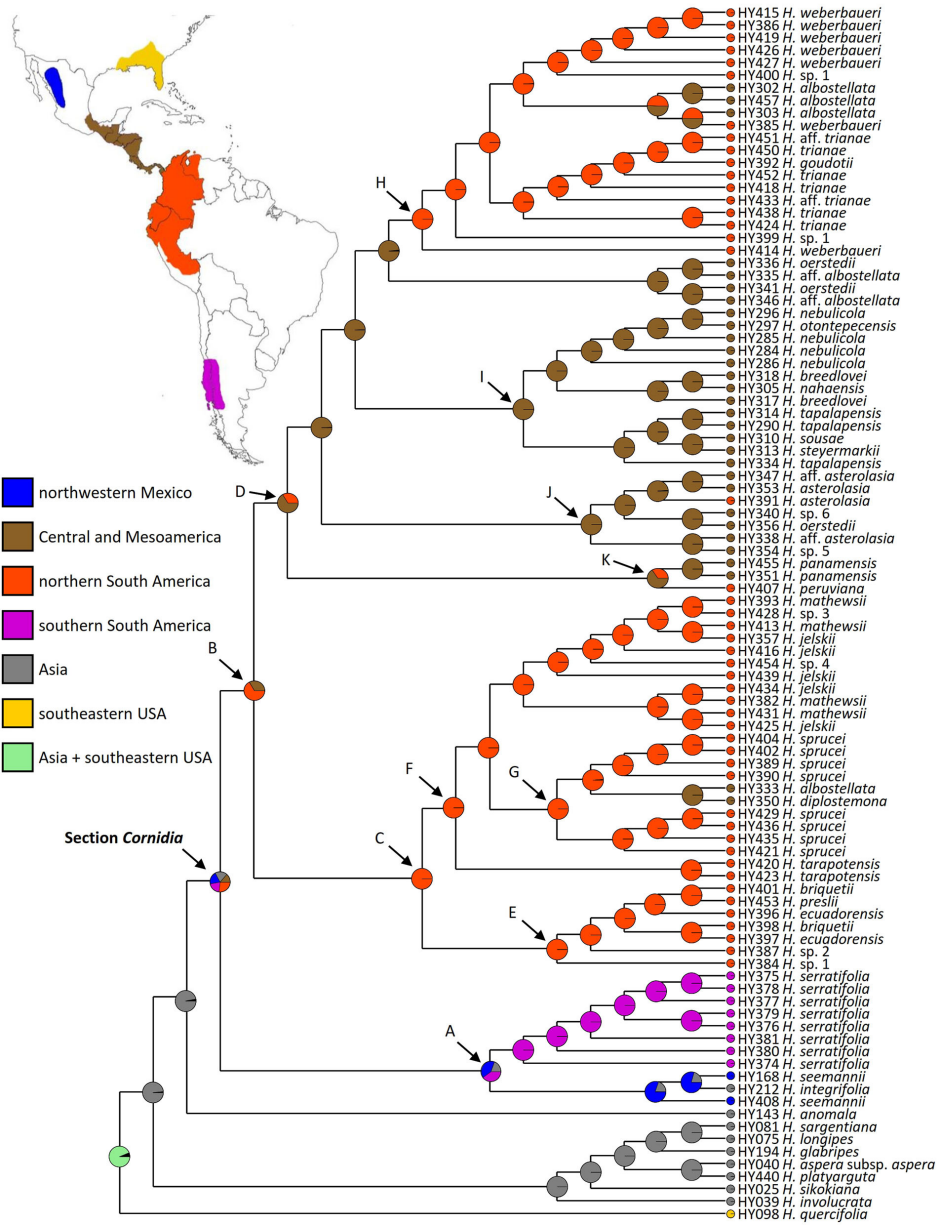
Ancestral area reconstruction analysis in BioGeoBEARS for *Hydrangea* section *Cornidia* under the Dispersal–Vicariance Analysis with long-distance dispersal (DIVA+J) model.

The Dispersal–Vicariance Analysis with long-distance dispersal (DIVA+J) was selected as the best fitting model for geographic range (AICc = 0.7; p < 0.01). This model recovered Asia as the most probable (0.97) ancestral area for the node subtending sections *Cornidia* and *Calyptranthe*, whereas for the node subtending *Cornidia* Asia was only recovered with 19% probability and ca. 80% in the New World. Within the New World, probabilities were distributed as follows: northwestern Mexico (ca. 20%), Central and Mesoamerica (ca. 13%), northern South America (ca. 27%), and southern South America (ca. 20%; Figure 6). Ancestral area probabilities for main clades recovered within *Cornidia* are shown in Table 1.

**Table 1:** Reconstructed ancestral area probabilities for main clades within *Hydrangea* section *Cornidia*. Node codes correspond to those used in Figure 6.

	**Node codes**										
	**A**	**B**	**C**	**D**	**E**	**F**	**G**	**H**	**I**	**J**	**K**
Northwestern Mexico (a)	0.4005	–	–	–	–	–	–	–	–	–	–
Central and Mesoamerica (b)	–	0.3333	–	0.6666	–	–	–	–	1	0.9999	0.6580
Northern South America (c)	–	0.6666	1	0.3334	1	1	0.9998	0.9999	–	–	0.3420
Southern South America (d)	0.4057	–	–	–	–	–	–	–	–	–	–
Asia (e)	0.1937	–	–	–	–	–	–	–	–	–	–
Southeastern United States of America (f)	–	–	–	–	–	–	–	–	–	–	–
Hidden posterior probabilities	0.0002	0.0001	–	–	–	–	0.0002	0.0001	–	0.0001	–

## Discussion

### Former Subdivisions of Section *Cornidia* in the Light of a First Molecular Phylogenetic Hypothesis

Briquet (1919) classified all species of *Cornidia* in two subsections, each with their respective series. Subsection *Monosegia* Briq. was divided in two series, namely, *Speciosae* Briq. and *Aphananthae* Briq., whereas subsection *Polysegia* Briq. contained the series *Synstyleae* Briq. and *Chorystyleae* Briq. Subsection *Monosegia* is characterized by a single pseudo-umbellate cyme and its series by the presence or absence of “sterile flowers,” respectively. However, it should be noted that the latter flowers are not sterile but have enlarged sepals, and hence, we prefer the term enlarged “marginal flowers,” following Samain et al. (2014, 2021). Subsection *Polysegia*, according to Briquet (1919), is recognized by the thyrsoid inflorescence, consisting of several pseudo-umbellate cymes, and its series, among others, by short stamens and pubescent leaves or long stamens and glabrous leaves. Subsection names defined by Briquet (1919) were later adopted by McClintock (1957), although with amended morphological characteristics of inflorescence architecture. Subsection *Monosegia* is characterized by opposite umbellate cymes, each in the axil of a leaf, and subsection *Polysegia* is recognized by the robust thyrsoid inflorescence, consisting of several umbellate cymes. Although the morphological classification in subsections according to Briquet (1919) and McClintock (1957) may seem valid at first sight, our own field and herbarium studies revealed specimens of several species with both inflorescence types, such as *Hydrangea* sp. 4, and of a yet undescribed species from Ecuador, indicating that these subsections are not natural. In the case of subsection *Monosegia*, we have observed that, although the enlarged marginal flowers generally characterize specific species, they may also be absent in some individuals of those species. In contrast, in species that generally do not possess them, they may be present, reduced in size or parts, or not reduced. With respect to the series of subsection *Polysegia*, the stamen length distinguishes between both sexes within a dioecious species: male individuals have long filaments, whereas these are short in female individuals. Indeed, our molecular phylogenetic results confirm the non-monophyly of the subsections, as well as the respective series (Figure 2). In addition, some species discussed in the next section are not monophyletic and will thus require further research (Figure 2). Based on the currently available data, we do not have sufficient elements to further subdivide *Cornidia* into subsections. However, some clades can be characterized by morphological and geographical patterns.

### Monophyly of Morphologically Defined Section *Cornidia* Species

As a result of our extensive field and herbarium studies, several new *Cornidia* species were discovered or existing species circumscriptions were amended (Samain et al., 2014, 2019, 2021). Our molecular phylogenetic hypothesis allows us to test the monophyly of many morphologically defined taxonomic entities for which more than one accession could be included (Figure 2). However, statistical support on species level is low in general. Only three species were recovered with high support as monophyletic, namely *H. panamensis* Standl., *H. serratifolia* (Hook. and Arn.) F. Phil. and *H. tarapotensis* Briq., whereas statistical support for some shallow nodes did not allow to refute, with high support, the monophyly of several of the herein recognized species.

Monophyletic *H. panamensis* is sister to the single accession available of *H. peruviana* Moric. ex Ser. Until recently, *H. peruviana* was considered as the most widespread *Hydrangea* species in the Neotropics; however, we now know that it is very rare and endemic to Ecuador (Samain et al., 2021). *Hydrangea panamensis* and *H. peruviana* can be separated based on striking leaf characters, including the shape and distribution of their acarodomatia, a peculiar symbiotic syndrome to accommodate mites on the abaxial leaf side. Actually, the presence of acarodomatia is common in Neotropical *Cornidia* species, but this has not been recorded in studies previous to the start of our research. Recent studies have shown that acarodomatia morphology and their distribution on the abaxial leaf surface are of taxonomic value in this group (Samain et al., 2014, 2019, 2021).

*Hydrangea serratifolia* from Chile was recovered as sister to a clade that includes *H. seemannii* L. Riley and *H. integrifolia*. Although these three species have white flowers, *H. serratifolia* differs from the other two in being dioecious (vs. monoecious) and lacking enlarged marginal flowers (see sections Sexual Dimorphism and Floral Characters in *Cornidia* and Unusual Disjunct Distribution in Section *Cornidia*). The last monophyletic species is *H. tarapotensis*. Previous studies have synonymized *H. tarapotensis* from Peru with *Hydrangea bangii* Engl. from Bolivia and *H. antioquiensis* Engl. from Colombia, based on the superficial similarities of their branched inflorescences. However, a detailed study of the type specimens of all three species showed that they can be easily distinguished based on leaf and inflorescence morphology (Samain and Martínez Salas, own observations). Neither *H. bangii* nor *H. antioquiensis* could be included in our taxon sampling due to the lack of material of sufficient DNA quality.

Five morphologically defined taxa were recovered with high support as non-monophyletic, including *H. albostellata* Samain, Najarro and E. Martínez; *H*. aff. *albostellata*; *H. breedlovei* Samain, Najarro and E. Martínez; *H. oerstedii* Briq.; and *H*. sp. 1. In the case of the Mesoamerican *H. albostellata*, only four Mexican accessions could be included. Three out of four of these accessions were recovered within clade H together with other Ecuadorian and Peruvian species. The fourth Mexican *H. albostellata* was retrieved as sister to a second Mesoamerican species, *H. diplostemona* Standl. from Costa Rica, which was nested within clade C, which is also entirely composed of Peruvian and Ecuadorian species. Apart from Mexico, specimens of *H. albostellata* have been collected from Honduras, El Salvador, Nicaragua, and Costa Rica (Samain et al., 2014). Given the ample geographic distribution of *H. albostellata* within Mesoamerica, the genetic exchange could occur across the entire *H. albostellata* distribution area, along with genetic exchange between Central American *H. albostellata* individuals and *H. diplostemona* from Costa Rica. Within clade D, there are twice a *H. oerstedii* accession and a *H*. aff. *albostellata* accession in a sister relationship. In both groups, the respective sister accessions grow in close proximity to each other in Costa Rica, suggesting an ongoing exchange of genetic material. The non-monophyly of *H. breedlovei* results from the position of the single *H. nahaensis* Samain and E. Martínez accession that we could include in our sampling. Both species are endemic to the Mexican state of Chiapas and can be easily distinguished by leaf shape and pubescence, as well as inflorescence morphology and whole plant architecture (Samain et al., 2019). Actually, *H. breedlovei* and *H. nahaensis* are recovered within clade I that is entirely composed of Mexican species, i.e., the recently described *H. otontepecensis* Samain and E. Martínez, *H. sousae* Samain, Najarro and E. Martínez, and *H. tapalapensis* Samain, Najarro, and E. Martínez (Samain et al., 2019), as well as *H. nebulicola* (Nevling and Gómez Pompa) and *H. steyermarkii* Standl. The latter was originally only known from Guatemala but has recently been discovered in Mexico as well (Samain et al., 2014). Species within clade I are characterized by simple inflorescences with white flowers and without enlarged marginal flowers and are easily distinguishable from other species based on plant architectural characters, branch and leaf pubescence, and leaf features such as shape, veins, and acarodomatia (Samain et al., 2019).

An additional finding is that a yet undescribed morphological entity, here preliminary called “*H*. sp. 1,” appears in distantly related clades (clades H and E; Figure 2). This indicates the need for the revision of the morphological characters used to circumscribe this entity, once more material than the one we currently dispose of becomes available. Within clade E, one accession of *H*. sp. 1 is grouped together with two accessions, each of *H. briquetii* Engl. and *H. ecuadorensis* Briq., as well as the only accession of *H. preslii* Briq. and *H*. sp. 2. *Hydrangea briquetii* and *H. ecuadorensis*, had been synonymized with *H. preslii* by McClintock (1957). Within clade H, two accessions of *H*. sp. 1 are related to multiple accessions, each of *H. weberbaueri* Engl., *H. albostellata, H. trianae* Briq., and *H*. aff. *trianae*, as well as a single accession of *H. goudotii* Briq.

The present phylogenetic framework will enhance our ongoing taxonomic revision of *Cornidia*, as it enables us to describe and discuss species in an evolutionary framework, which is especially helpful, given their nomenclatural and morphological complexity. Vice versa, correct identification of all accessions in the current sampling would not have been possible in the absence of the more than 10 years of experience acquired by working in the field and with herbarium specimens of this very intricate group. It is worth mentioning that the initial identification we used for our accessions mainly consisted of the 11 then used names, completed with a range of morphospecies we had detected, resulting in a confuse pattern of names, especially in South America. Our careful study of all types of specimens of *Cornidia* species and comparison with our own collections and herbarium specimens have been essential to solve this puzzle. On the one hand, we realized that the currently accepted circumscription of some taxa is erroneous, and that, on the other hand, an important number of the morphological variation, in fact, already had been delimited in valid species, but these had been synonymized and just considered as some aberrant forms of widespread species (Samain et al., 2019, 2021).

Much work is still needed in order to present a fully coherent species concept that is reflecting natural relationships. Complementary studies, including multiple individuals per morphological species, along with a next-generation sequencing approach, such as the RADseq strategy applied to *Hydrangea* section *Asperae*^1^, will be useful to further inform species circumscription within *Cornidia*.

### Sexual Dimorphism and Floral Characters in Section *Cornidia*

**Figure 7:**
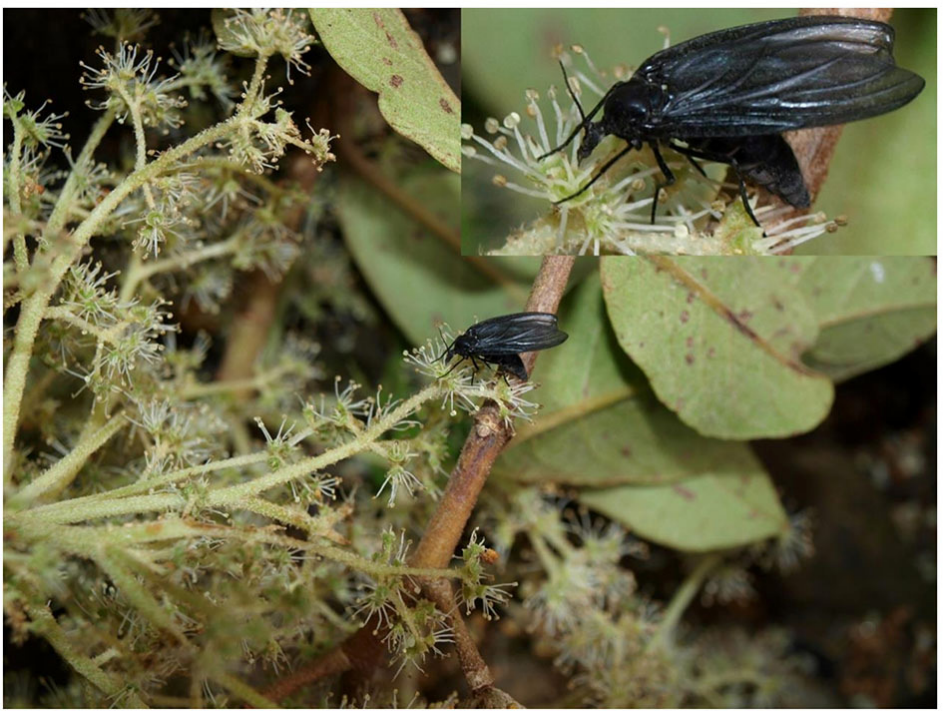
An unknown fungus gnat species belonging to the genus *Sciara* (Sciaridae) visiting the flower nectaries of *Hydrangea jelskii* (Samain et al., 2011-059, Chachapoyas, Amazonas, Peru).

To date, there is only very limited knowledge about *Hydrangea* reproductive biology (Reed, 2005), as is the case for *Cornidia* representatives. During own fieldwork in Peru, we observed many individuals of an unknown fungus gnat species belonging to the genus *Sciara* (Sciaridae), visiting the flower nectaries of *H. jelskii* (Figure 7). However, it is yet unknown whether these flies, indeed, are attracted because of the food source or in search of breeding partners. Also, the sex of the flies could not be determined. For *H. serratifolia* in Chile, Smith-Ramírez and coauthors (2005) reported potential pollinators from Diptera (Syrphidae) and Hymenoptera (Apidae, Halicitidae, and Vespidae). In *H. serratifolia*, strong dependence on Hymenoptera was concluded, and that pollen is used as a food source (Smith-Ramírez et al., 2005).

Nevling and Gómez-Pompa (1968) first documented dioecism in *Cornidia*. Within *Hydrangea, Cornidia* is the only section for which dioecism has been documented (Freire-Fierro, 2004; Samain et al., 2014, 2019, 2021; Samain and Martínez Salas, 2015). This sexual dimorphism can be described as functional, as both female and male structures are present, although male structures are reduced in size and not functional in female plants, and vice versa. The presence of dioecy in most *Cornidia* species is remarkable since this condition is known to be rare, although well-distributed across angiosperm lineages (Renner, 2014). Dioecious lineages within hermaphrodite genera are, for instance, known in Lamiaceae (*Lepechinia* section *Parviflorae*; Drew and Sytsma, 2013), although, in this case, also including gynodioecious populations. Among genera entirely composed of dioecious species, the majority seem to have lower species richness (Renner, 2014). Future studies could formally test if transitions between these sexual morphs are associated with changes in diversification rates in *Cornidia*. Our character reconstruction of sexual dimorphism clearly shows that dioecism is the ancestral state in *Cornidia* (Figure 3) and that this was reversed to monoecy in *H. seemannii* and *H. integrifolia*. Henry et al. (2018) mentioned that lineages having a dioecious ancestral condition and presenting hermaphroditic descendants are rare as well. Reversal to hermaphroditism among individuals within a species population has been documented (Ehlers and Bataillon, 2007); however, the potential of such hermaphroditic individuals to evolve into a new species has not been demonstrated (Henry et al., 2018).

We found no indication that dioecy is correlated with any of the other two evaluated floral characters. Drew and Sytsma (2013) found a correlation between flower color and dioecy in their study of *Lepechinia* (Lamiaceae); however, a range of colors is observed in the dioecious *Cornidia* species. The same applies to the presence/absence of enlarged marginal flowers as this seems to vary freely within *Cornidia*.

Enlarged marginal flowers are well-known from cultivated *Hydrangea* species. This character state is widely distributed in this genus in general. This is in line with the results of our floral character reconstruction that shows, with higher probability, the presence of enlarged marginal flowers as the ancestral character state in *Cornidia* (Figure 4). However, the minority of *Cornidia* species exhibit this character state. At least three *Cornidia* clades independently lost enlarged marginal flowers, and losses are reconstructed to be more likely than their gain. This is also in accordance with several independent losses reconstructed within clade H (Figure 4). In field experiments carried out in Japan, it has been shown that enlarged marginal flowers (called “decorative flowers” in the original publication) increased the frequency of floral visitors and that decorative flowers act as landing sites of visitors (Wong Sato and Kato, 2019). The latter study concluded that the main function of decorative flowers is to increase pollinator attraction by an increasing inflorescence surface.

Besides the enlarged marginal flowers of *Hydrangea*, about whose function very limited knowledge is available, only few other angiosperm lineages show comparable functional morphological structures that are sepal or petal derived. As reviewed by Ohashi and Yahara (2001), one of the reasons for producing these structures is to enlarge the display to attract floral visitors. The genus *Viburnum* (Adoxaceae) exhibits enlarged marginal, petal-derived sterile flowers (Lu et al., 2017). This genus is also not producing large amounts of nectar, and the nectar is only produced for a short period of time (Donoghue, 1983). Although unknown for the species in *Cornidia*, a similar investment in floral structure while reducing pollinator reward might be the driving force for the observed floral phenotypes to maintain or increase reproductive success. Experimental data obtained from *Viburnum* also suggest enhanced reproductive success through effectively attracting pollinators and thus enhancing a fruit set (Jin et al., 2010). Manipulation experiments in *Viburnum*, removing the enlarged marginal flowers, confirm the reduced pollinator attraction and female reproductive success (Park et al., 2019). Wong Sato and Kato (2019) also showed this for wild *Hydrangea* species in Japan and confirmed the enlarged marginal flowers to boost attractiveness to pollinators. If this is also true for *Cornidia* species can yet only be speculated as no experimental data are available.

Other examples of enlarged marginal flowers with enlarged sepals (one per flower only) can be found in the genera *Calycophyllum* and *Cosmocalyx* of the family Rubiaceae. The inflorescences of the representatives of the first genus are morphologically very similar to *Hydrangea*, but literature on its pollination syndrome is contradictory (ranging from hummingbirds over butterflies to wind pollination, the latter being very unlikely, given the flower morphology, e.g., Hilje et al., 2015), whereas the slightly enlarged sepal in species of the second genus continues to grow during fruit development to play a role in dispersal (Delprete, 1998). Similar enlarged marginal flowers with enlarged petals, apart from *Viburnum*, can be found, e.g., in the families Apiaceae and Asteraceae, well-known for the development of pseudanthia or pseudo-flowers.

Flower color polymorphisms are generally attributed to three major groups of pigments other than chlorophyll, i.e., betalains, anthocyanins, and carotenoids (Grotewold, 2006; Lagorio, 2013; Nadot and Carrive, 2021). Anthocyanins are most widespread in angiosperms (Grotewold, 2006) and change unpigmented white flowers to a diverse mosaic of colors, including yellow, orange, red, pink, purple, or blue (Zhao and Tao, 2015; Nadot and Carrive, 2021). Besides the pigmentation itself, transposable element activity is well-known to change floral color as well, such as in *Ipomoea* (Clegg and Durbin, 2000). The blue/pink pigmentation of *H. macrophylla* flowers has been studied intensively (Yoshida et al., 2003; Oyama et al., 2015; Ito et al., 2019; Peng et al., 2021). More recently, Yoshida et al. (2021) provided experimental evidence for cell color variation in *Hydrangea macrophylla* sepals where the anthocyanin, 3-*O*-glycosyldelphinidins, three co-pigment components, acylquinic acids, and aluminum ions (Al^3+^) result in graded differences from red, through purple, to blue, even in neighboring cells within a single sepal. In *Cornidia*, either unpigmented sepals are observed or the whitish to reddish color phenotypes, indicating that a similar cell mosaic, as known from *H. macrophylla* (Yoshida et al., 2021), might play a role in gradual flower coloration as well. The main function of flower color is the attraction of pollinators. It is largely unclear which insects (potentially other pollinators as well, see above) are acting as pollinators in *Cornidia*, and thus it is yet unclear whether observed color phenotypes are discriminating specific pollinators while attracting others. In the majority of the *Cornidia* species, only a single coloration is observed (red), with the exception of *H*. sp. 1 and *H. sprucei*. We can likely exclude a sampling artifact, as we have never observed color changes when revisiting individual locations. Certainly, flower coloration among populations of a single species will need further studies in *Cornidia*.

### Unusual Disjunct Distribution in *Cornidia*

The genus *Hydrangea* is generally characterized by a disjunct distribution between Asia and America. Apart from *Cornidia*, a few *Hydrangea* species occur in the eastern USA, whereas the majority are distributed in eastern Asia (McClintock, 1957; Samain et al., 2010; De Smet et al., in review). The most likely ancestral area for the node subtending sections *Cornidia* and *Calyptranthe* is Asia (ca. 97% probability), although the origin of *Cornidia* is the New World (ca. 81% New World vs. 19% Asia, Figure 6). However, within the New World, several potential ancestral areas, including northwestern Mexico (ca. 20%), Central and Mesoamerica (ca. 13%), northern South America (ca. 27%), and southern South America (ca. 20%) are reconstructed.

Within clade A, three species are grouped together that span the extremes of the *Cornidia* distribution area, i.e., the northwestern Mexican *H. seemannii*, the southern south American *H. serratifolia*, and the Asian *H. integrifolia* (Figure 6). The ancestral area of clade A is reconstructed to be northwestern Mexico, southern South America, and Asia (ca. 40, 40, and 20% probability, respectively). On the one hand, two not necessarily exclusive options might explain these results: (1) at some point in time, *Cornidia* species had a broad distributional area, including the extremes of today's distribution, and/or (2) a second long-distance dispersal event which has to be assumed from the New World to Asia. However, given the morphological similarity between *H. seemannii* and *H. integrifolia*, as opposed to *H. serratifolia*, it seems most likely that the ancestor of the Asian species came from the New World. On the other hand, a broad ancestral area is supported by the available *Hydrangea* leaf fossil record from Chile from Late Eocene deposits (Otero et al., 2012), as well as the Oligocene *Hydrangeiphyllum*; (Friis and Skarby, 1982). *Hydrangea seemannii, H. integrifolia*, and *H. serratifolia* occur at temperate or subtropical latitudes in more temperate climate, between 900 and 2,400 m in Taiwan and the Philippines, temperate forests of Chile and Argentina, and between 2,500 and 2,900 m in northwestern Mexico, respectively (Baeza et al., 1999; Arroyo et al., 2000, p. 200; Samain and Martínez Salas, 2015; https://www.philippineplants.org/index.html).

Within clade B, a New World ancestral area is reconstructed with northern South America and Central- and Mesoamerica as the most probable areas of distribution (ca. 66 and 33%, respectively), although only a single species, i.e., *H. asterolasia* Diels, which is known from Costa Rica and Ecuador, is shared between the two adjacent areas (Freire-Fierro, 1999, 2004; Morales, 2007; Monro et al., 2017). The ancestor of clade C is clearly reconstructed as northern South America (100% probability), whereas clade D and the majority of successive lineages are clearly reconstructed as Central- and Mesoamerica ancestral areas with at least three reversals between Central- and Mesoamerica and northern South America and vice versa (Figure 6). Since the arrival of the *Cornidia* ancestor in the New World, significant diversification occurred that today is the second most species-rich area for *Hydrangea* in general after eastern Asia.

Our ancestral area reconstruction results and the fact that *Hydrangea* species have dust-like diaspores that could be dispersed by wind, water, or on animal bodies (Armesto and Rozzi, 1989; Hufford, 1995) suggest that the ancestor of the section arrived in the New World by long-distance dispersal from Asia. In contrast to the well-known North American—East Asian disjunction (e.g., Graham, 1972; Axelrod, 1975; Cracraft, 1975; Donoghue et al., 2001; Wen et al., 2010; González et al., 2014; Feng et al., 2020), the unusual Asian/Central- and Mesoamerican—South American disjunction, which is not including eastern North America, is less well-known in plants and has only been investigated recently (Wang et al., 2020; Zhu et al., 2020; Yao et al., 2021). These authors, respectively, in Lardizabalaceae, *Celastrus* (Celastraceae), and *Ilex* (Aquifoliaceae), hypothesize an eastern Asian origin of the taxon studied, followed by one or more long-distance dispersal events to Central and South America (and in the case of *Celastrus* also to other continents), showing this pattern to be more common than previously assumed, thus strengthening our own hypothesis.

Looking at the overall distribution pattern of the family Hydrangeaceae and the genus *Hydrangea s.l*. (North, Central, and South America and eastern and southern Asia), several other studies can help us to understand or explain the current disjunct distribution, even in the absence of fossil *Hydrangea* records. Azuma et al. (2001), in their dated molecular phylogeny of *Magnolia* (Magnoliaceae), whose distribution is very similar to *Hydrangea*, show, albeit with a limited sampling of Neotropical species, the relictual tropical Asian and American *Magnolia* disjunctions to be a result of the expansion of the circumboreal tropical flora from the middle to the end of the Eocene, followed by cooling temperatures causing the tropical disjunctions, indirectly confirmed by a dated phylogenetic study, including all Caribbean *Magnolia* species and a small sample of Neotropical taxa^2^. Similarly, Antonelli and Sanmartín (2011), in their reconstruction of the spatiotemporal evolution of *Hedyosmum* (Chloranthaceae), which, apart from the very similar distribution pattern, also shows similar species numbers (one Paleotropical and 44 Neotropical), hypothesize that the extant diversity is only a remnant of past radiation, which has suffered high extinction rates.

## Conclusions

We present the first molecular phylogenetic hypothesis of *Cornidia*, including a dense and highly representative taxon sampling. This group of lianas occurs from northern Mexico to southern Chile and Argentina, with one species in Southeast Asia only. Besides that, we proved the non-monophyly of traditionally recognized infrageneric taxa entities. We also showed that some morphological species do not seem to be monophyletic. Furthermore, we reconstructed the evolution of selected reproductive characters, as well as the biogeography of this section. This study clearly shows that more research is needed, apart from the continuation of the taxonomic review of the whole section. Future studies could formally address whether shifts between dioecy and hermaphroditism could have influenced speciation in *Cornidia*, as well as to assess the genetic basis of the transition between these two sexual systems in the group. Additionally, the roles that climatic and orogenic factors have played in the diversification of the group are yet to be explored. Future research could aim at performing a nuclear enrichment strategy in combination with genome skimming to generate, in parallel, both uniparental (i.e., plastid genomes and mitochondrial scaffolds) and biparental data (nuclear single or low copy regions) to increase phylogenetic resolution at shallow depths. This information could afterward be used to test stages of evolutionary radiation (Naciri and Linder, 2020) in *Cornidia*.

## Data Availability Statement

NCBI accession numbers for the sequence data associated to this article can be found in the Supplementary Material 1.

## Author Contributions

M-SS, SW, and PG conceived the project. CG, EM, and M-SS realized fieldwork. CG and M-SS designed the sampling. EM and M-SS identified the accessions. M-SS coded floral characters. CG, SW, and M-SS coded the biogeographical areas. CG and SW realized lab work and analyzed the data. CG, EM, SW, and M-SS wrote the manuscript. All authors revised and approved the final version.

## Conflict of Interest

The authors declare that the research was conducted in the absence of any commercial or financial relationships that could be construed as a potential conflict of interest.

## Footnotes

^1^De Smet, Y., Cires, E., De Clerck, O., Goetghebeur, P., Wanke, S., and Samain, M.-S. (in review). Genome-wide RADseq data resolves phylogeny and species boundaries in the *Hydrangea aspera* species complex. *Mol. Phyl. Evol*.

^2^Veltjen, E., Testé E., Palmarola Bejerano, A., Ortiz, R. C., Jabbour, F., and Chen, Z.-D. (in review). The evolutionary history of the Caribbean Magnolias (Magnoliaceae): testing species delimitations and biogeographical hypotheses using molecular data. *Mol. Phyl. Evol*.

## Supplementary Material

Supplementary Material 1 Taxon sampling with voucher information, botanical garden accession numbers where applicable, field data, and GenBank accession numbers.

Supplementary Material 2 Original alignment and the alignment used for phylogenetic inference where an ambiguously aligned region was excluded.

Supplementary Material 3 Individual *SMC1* and *TIF3H1* gene trees.
