# Supplementary figures and images for: Molecular Phylogeny, Character Evolution, and Biogeography of Hydrangea Section Cornidia, Hydrangeaceae
Source: Front Plant Sci. 2021 Jun 29;12:661522. doi: 10.3389/fpls.2021.661522 (PMC8276264; doi:10.3389/fpls.2021.661522)

SMC1

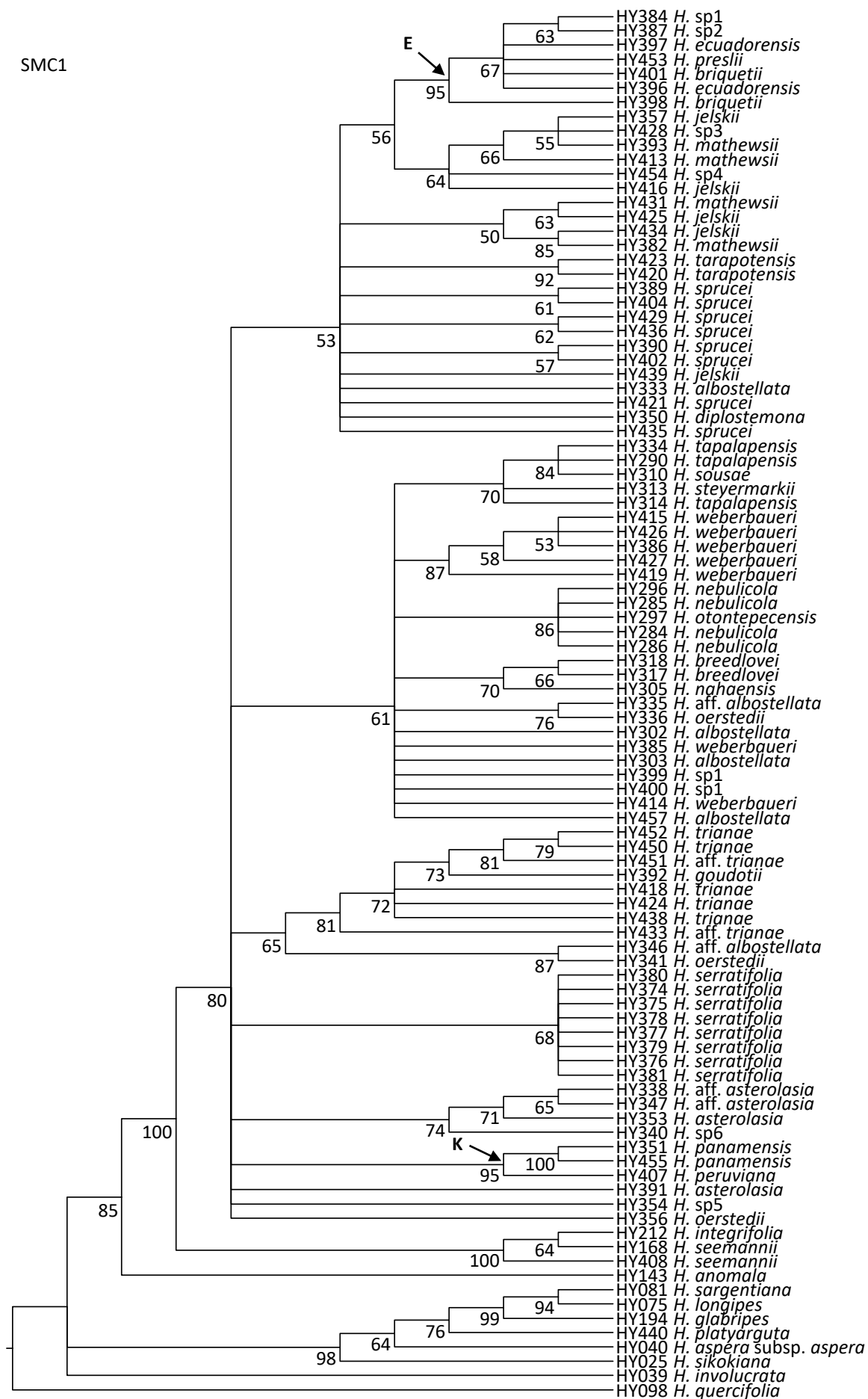

Supplement: Supplementary Material 1 — Taxon sampling with voucher information, botanical garden accession numbers where applicable, field data, and GenBank accession numbers. [file Data_Sheet_1.zip › Supplementary Material S3_SMC1.pdf]

TIF3H1

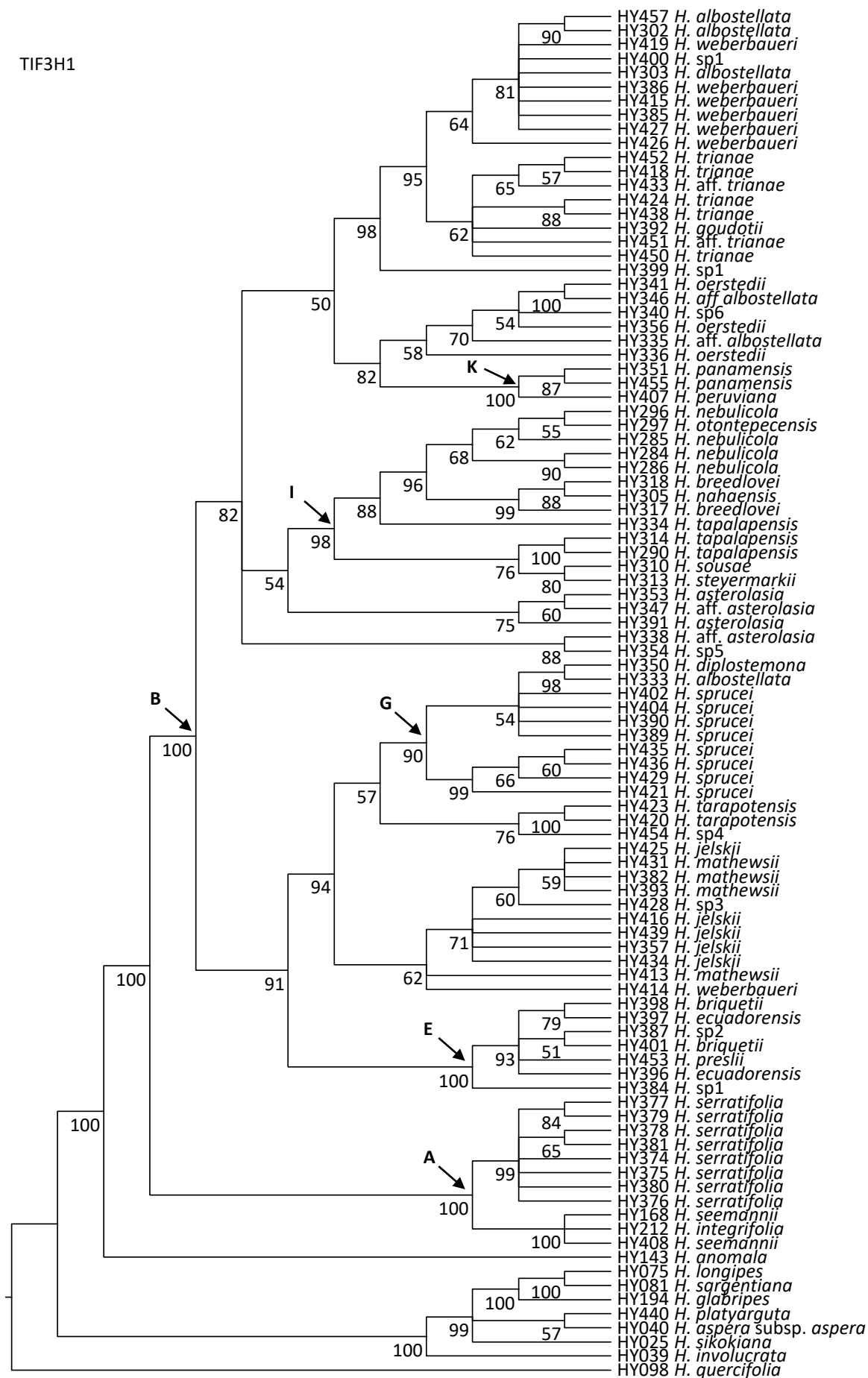

Supplement: Supplementary Material 1 — Taxon sampling with voucher information, botanical garden accession numbers where applicable, field data, and GenBank accession numbers. [file Data_Sheet_1.zip › Supplementary Material S3_TIF3H1.pdf]
